# Endothelial-like properties of claudin-low breast cancer cells promote tumor vascular permeability and metastasis

**DOI:** 10.1007/s10585-013-9607-4

**Published:** 2013-08-22

**Authors:** J. Chuck Harrell, Adam D. Pfefferle, Nicole Zalles, Aleix Prat, Cheng Fan, Andrey Khramtsov, Olufunmilayo I. Olopade, Melissa A. Troester, Andrew C. Dudley, Charles M. Perou

**Affiliations:** 1 Lineberger Comprehensive Cancer Center, University of North Carolina at Chapel Hill, Campus Box 7295, 450 West Drive, Chapel Hill, NC 27599 USA; 2 Department of Genetics, University of North Carolina at Chapel Hill, Chapel Hill, NC 27599 USA; 3 Department of Pathology & Laboratory Medicine, University of North Carolina at Chapel Hill, Chapel Hill, NC 27599 USA; 4 Department of Epidemiology, University of North Carolina at Chapel Hill, Chapel Hill, NC 27599 USA; 5 Department of Cell Biology and Physiology, McAllister Heart Institute, University of North Carolina at Chapel Hill, Chapel Hill, NC 27599 USA; 6 Translational Genomics Group, Vall d’Hebron Institute of Oncology (VHIO), Barcelona, Spain; 7 Section of Hematology/Oncology, Department of Medicine, The University of Chicago, Chicago, IL 60637 USA

**Keywords:** Angiogenesis, Breast cancer, Gene signature, Metastasis, Microarray

## Abstract

The vasculature serves as the main conduit for breast tumor metastases and is a target of therapeutics in many tumor types. In this study, we aimed to determine if tumor-associated vascular properties could help to explain the differences observed in metastagenicity across the intrinsic subtypes of human breast tumors. Analysis of gene expression signatures from more than 3,000 human breast tumors found that genomic programs that measured vascular quantity, vascular proliferation, and a VEGF/Hypoxia-signature were the most highly expressed in claudin-low and basal-like tumors. The majority of the vascular gene signatures added metastasis-predictive information to immunohistochemistry-defined microvessel density scores and genomically defined-intrinsic subtype classification. Interestingly, pure claudin-low cell lines, and subsets of claudin-low-like cells within established basal-like cancer cell lines, exhibited endothelial/tube-like morphology when cultured on Matrigel. In vivo xenografts found that claudin-low tumors, but not luminal tumors, extensively perfused injected contrast agent through paracellular spaces and non-vascular tumor-lined channels. Taken together, the endothelial-like characteristics of the cancer cells, combined with both the amount and the physiologic state of the vasculature contribute to breast cancer metastatic progression. We hypothesize that the genetic signatures we have identified highlight patients that should respond most favorably to anti-vascular agents.

**Electronic supplementary material:**

The online version of this article (doi:10.1007/s10585-013-9607-4) contains supplementary material, which is available to authorized users.

## Introduction

There are at least five genomically distinct subtypes of human breast tumors [1, 2]. Each tumor subtype interacts with endothelial cells (EC) via secreted factors and directly by cell–cell contact (reviewed in [3]). These associations facilitate cancer cell entry into blood- and lymphatic-vessels, which initiates the metastatic cascade and results in the death of ~400,000 people worldwide each year [4]. The dependence of primary tumor growth on angiogenesis was first proposed over 40 years ago [5], and since then the amount of histologically defined vasculature within a tumor has been shown to be correlated with tumor metastatic potential in nearly all solid cancer types [6–8].

We recently reported that the estrogen receptor (ER) negative basal-like and claudin-low tumor subtypes are likely to spread to vital organs such as the brain and lung; without targeted therapies, HER2-enriched tumors aggressively colonize the liver, while ER+/luminal A and luminal B tumors are slower to disseminate and are usually first identified in the bone [9]. It is possible that some breast cancer subtypes are predisposed to metastasize more readily than others due to the amount of vasculature present within and surrounding the primary tumor. Therefore, we hypothesized that basal-like and claudin-low tumors, as compared to luminal tumors, preferentially attract increased numbers of blood- and lymphatic-endothelial cells (BEC, LEC, collectively EC), which facilitates their metastasis via vessel association, intra/extravasation and dissemination. Alternatively, there may be no major difference in the amount of vasculature present within different subtypes, but instead, the physiologic properties of the cancer cells regulate how different subtypes interact with ECs.

To identify vascular contributions to breast tumor metastatic progression, we identify and contrast multiple distinct EC gene expression programs in comparison with known clinical variables and other published genomic signatures. We have found vascular gene expression signatures that add metastasis-predicting information to pathologically-defined microvessel density scores. In addition, we also find that the different breast cancer subtypes not only vary in their expression of endothelial genes, but further, that these genomic programs result in permeability of the vasculature in claudin-low tumors.

## Materials and methods

### Cell culture and imaging experiments

The human breast cancer cell lines were maintained in standard growth media (ATCC: MCF-7, T47D, SKBR3, MDA231) in RPMI (Gibco) plus 10 % FBS (Sigma) and PenStrep (Gibco); Asterland SUM149, SUM159 in Ham’s F-12 (Gibco) plus 5 % FBS (Sigma), insulin (Gibco 5ug/mL), and hydrocortisone (Gibco 1ug/mL). Human endothelial cells (EC) were all purchased from Lonza and were grown in EBM-2 media with BulletKit additives (Lonza CC-3202). Blood microvascular endothelial cells (BEC) and lymphatic microvascular endothelial cells (LEC) were from the same donor, and along with HUVECs, were all used within 5 passages. All vascular cell lines were grown in EBM-2 for 48 h prior to RNA extraction (monocultures and cocultures). Three-dimensional morphology experiments were performed by coating a Lab-Tek 8-well chamber slide (Thermo Scientific) with 125 uL of Matrigel (Becton–Dickinson) and then plating 50,000 cells in each well. To allow for discrimination of cancer cells and ECs, they were labeled with Sigma’s PKH67 (green) and PKH26 (red) dyes, respectively, prior to co-culture. All morphological studies (Fig. 4C–E) were performed for 18 h. Images of cell culture experiments were taken with a Nikon inverted phase contrast microscope and recorded with OpenLab software (Fig. 4; Supplemental Fig. 2). Confocal images (Fig. 4E) were taken with an Olympus FV 500 Confocal Laser Scanning Microscope and processed with Olympus FluoView software. Immunofluorescence images of xenografts tumors were acquired with an Olympus IX81 Inverted Light Microscope. All fluorescence images were combined with Image J v1.46.

### Gene expression microarrays and gene signatures

RNA was prepared from human breast cancer cell lines and ECs with Qiagen’s RNeasy mini kit. Gene expression microarrays were performed according to established protocols [10, 11], with all microarray data publicly available at the UNC microarray database (UNCMD) https://genome.unc.edu/. New microarrays have been deposited in the Gene Expression Omnibus under the accession number GSE37145, with previously published data available under GSE31870. Prior to analyses, the expression data were downloaded from the UNCMD, and the probes were filtered by requiring the Lowess normalized intensity values in both sample and control to be greater than 10 dpi and present on more than 70 % of microarrays. The normalized log_2_ ratios (Cy5 sample/Cy3 control) of probes mapping to the same Entrez gene ID were averaged and median centered to obtain the final dataset.

For the vascular content signature, RNA was prepared from human breast cancer cell lines (MCF7, T47D, SKBR3, SUM149, SUM159, MDA231), endothelial cells (HUVEC, LEC, BEC) and commercial RNA for human tissue was obtained from Ambion (brain; AM6050), (lung; AM7968), (liver; AM7960), (lymph node; AM7894), Clontech (bone marrow; 636591), and Biochain (bone marrow; R1234024-10). The breast cancer cell line arrays and the human organ arrays were compared against endothelial cell arrays from BEC, LEC, and HUVEC in a two-class SAM. Genes with high expression were used to generate this signature.

The activated endothelium signature was generated from microarrays of BEC and LEC RNA that had been grown in monoculture or transwell co-cultured with cancer cells. For transwell co-culture, 200,000 endothelial cells were plated into a well of a 6-well plate, then a transwell filter with 0.4 micron pores (Corning) was inserted into the wells and 200,000 cancer cells were added to the upper compartment in EBM-2. After 48 h the transwell inserts were removed and the endothelial cell RNA was extracted (Qiagen). For the activated endothelium signature a two-class SAM was performed on triplicate BEC and LEC arrays compared to arrays from BECs and LECs that had each been transwell cultured with the six breast cancer cell lines described above. Each tumor’s endothelial signature score was determined by averaging the log_2_ expression values for all genes in the signature (either 74 for vascular content, or 110 for activated endothelium) that were also found in the different test datasets. To separate out the proliferation component of the signature, all genes with a Pearson correlation value greater than 0.5 to a 11-gene proliferation signature [12] were considered proliferation related; due to the reduced number of genes present, this signature was not able to be divided in the merged 550 tumor dataset. Lastly, a mouse mammary tumor gene expression dataset was also examined that has been previously published [13] (GSE3165 and GSE27101).

### Statistics and data analysis

All statistical tests were performed with WinSTAT, R v2.15.1, and Cluster v3.0.

### In vivo tumor studies and immunofluorescence

All animal procedures were done under a protocol approved by the University of North Carolina Animal Care and Use Committee. To establish MCF-7, MDA-231, SUM159 tumors, 3 Nod scid gamma (NOD.Cg-Prkdc^scid^ IL2rg^tm1Wjl^/SzJ JAX^®^) mice for each tumor type were anesthetized with isoflurane, and one-million cells in 100 % Matrigel with growth factors (Becton–Dickinson) were injected into the lactiferous duct of the fourth (inguinal) mammary gland. For MCF-7 tumors, mice were also implanted with an estradiol-releasing silastic pellet as previously described [14]. MDA-231 and SUM159 tumors were grown for 18 days, MCF-7 tumors were grown for 24 days. The difference in growth times reflects the amount of time needed to extract similar sized tumors. All tumors were removed when they were ~7 × 7 mm. To label perfusing vasculature in vivo, mice were injected with 1 mg of Texas red-conjugated dextran (molecular weight 70,000; Invitrogen/Molecular Probes, Eugene, OR) diluted in PBS (5 mg/mL) and then euthanized 5 min later, as previously described [15]. Prior to being embedded and frozen in Optimal Cutting Temperature (OCT) Compound, tissues were fixed in 10 % formalin overnight, and then 30 % sucrose overnight. Tissues were then stored at −80 °C until they were cut into 9–10 micron thick sections. Primary antibodies utilized were from Dako (vWF (A0082)), Novus Biosciences (LYVE1 [NBP1-43411], vimentin [NBP2-12472], cytokeratin 19 [NB100-79916]), Novacastra (CD34 [NCL-L-END]), and Santa Cruz (PECAM [sc-101454]). Secondary antibodies were from Molecular Probes/Life Technologies (Goat anti-rabbit 488) and Jackson ImmunoResearch (donkey anti-rat FITC). Mounting media containing DAPI was from Invitrogen (P36931).

## Results

### Vascular genomic programs are highly expressed in claudin-low and basal-like tumors

**Fig. 1:**
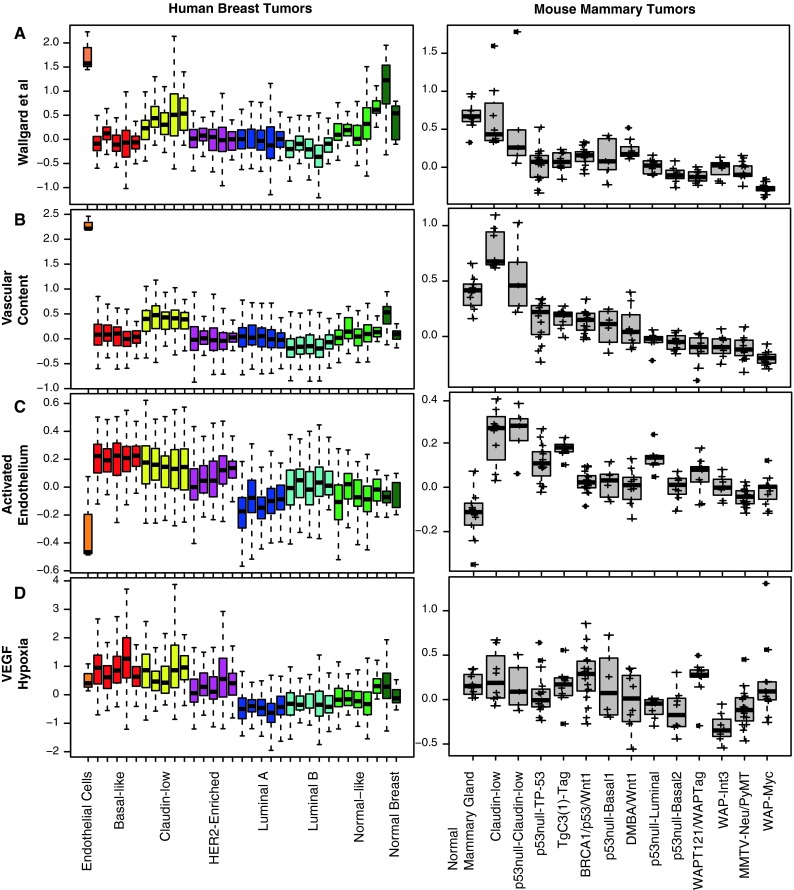
Vascular gene expression signatures in different intrinsic subtypes of human breast tumors and transgenic mouse models of mammary cancer. Box-and-whisker plots are shown for five human breast tumor datasets (*Left*) and a mouse mammary tumor dataset [13] (*Right*). Gene expression signature scores were identified for endothelial cell lines (BEC, LEC, HUVEC), each breast cancer dataset, and then combined for display in the following order within each subtype; Combined 855 [9], MDACC [20], Merged 550 [19], METABRIC [18], UNC [2]. **a** Wallgard et al., **b** vascular content, **c** activated endothelium, and **d** VEGF/hypoxia signatures. The log_2_ mean signature expression for each tumor is shown as a cross, the *bar* indicates the median value, *whiskers* show the range within subtype and are the 1.5 * inter-quartile range

The majority of nearly one-hundred publications have found that high microvessel density is associated with poor prognosis in breast cancer [16]. Since the results from this assay can vary depending on the vascular antibody utilized and/or one’s definition of a vascular hot-spot, we aimed to determine if vascular gene expression signatures could serve as an alternative biomarker to identify patients with an increased likelihood of distant metastasis or death. To test this hypothesis, we developed two novel Endothelial Cell (EC)-derived signatures and also tested a published EC signature that has been shown to be specific for the microvasculature [17]. For comparisons of these signatures, we used five previously published microarray data sets of breast cancer patients; these analyses comprised >3,000 human breast tumors with ~10 % overlap [2, 9, 18–20]. To contrast how the vascular signatures were expressed in normal breast samples, breast tumors, and pure endothelial cell lines, we determined average gene expression signature scores for each sample and in three EC lines (HUVEC, BEC, and LEC). To visualize these values across different subtypes of human breast tumors, normal breast samples, and EC lines, each sample’s signature was plotted as box-and-whisker plots (Fig. 1). On average, expression of the Wallgard et al. vascular signature was highest in EC lines, followed by normal breast reduction mammoplasty tissues (Fig. 1A). Interestingly, the claudin-low tumors had the highest vasculature signature expression when compared against any of the other tumor subtypes (*t* test, *p* < 0.0001). When this signature was examined on a database of mouse mammary tumors and normal mammary glands [13], the normal mammary tissue and murine claudin-low tumors also exhibited high expression (Fig. 1A).

We next generated a new vascular-specific signature that was designed to be completely distinct from mammary cells. We performed two-class significance analysis of microarrays (SAM) analyses on gene expression data from HUVECs, BECs, and LECs compared against organs that often harbor breast tumor metastases (bone marrow, brain, liver, lymph node, lung) and human breast cancer cells that represent multiple intrinsic subtypes (MCF-7, T47D, SKBR3, SUM149, SUM159, MDA-MB-231). This analysis identified 74 significantly upregulated genes in ECs (false discovery rate, FDR <0.05) **(**Supplemental Table 1). An Ingenuity pathway analysis of these genes identified the top biological functions to include cardiovascular system development, cancer, and cellular movement. This gene signature, hence forth called the ‘vascular content’ signature, is presented in Fig. 1B. This signature was also most highly expressed in claudin-low tumors as compared to other tumor subtypes (*t*-test, *p* < 0.0001), and compared to the Wallgard et al. signature this signature showed a larger separation between breast samples and ECs. The highest expression of the vascular content signature was observed in claudin-low mouse tumors (Fig. 1B). Interestingly, only two genes from our 74-gene vascular content signature overlapped with the 58 genes from Wallgard et al. (*EGFL7* and *ESAM*). We propose that the reason the normal breast samples have relatively higher levels of both the Wallgard et al. and vascular content signature are due to the relatively high endothelial content found in a normal breast.

We next sought to identify genes that change in ECs in response to signals from cancer cells. Thus, to evaluate a cancer-stimulated or ‘activated endothelium’ signature, we performed gene expression analyses on co-cultures of cancer cells and ECs. To identify common pathways involved in EC activation (independent of tumor subtype), we utilized different subtypes of cancer cells and generated a single signature of cancer cell-stimulated ECs. In these assays, the cancer cells and ECs exchanged secreted factors by being bathed in the same media, but both remained physically separated (see Methods). After 48 h of co-culture, a two-class SAM identified 110 genes that were significantly upregulated in ECs that had been transwell cultured with cancer cells as compared to ECs grown in monoculture (Supplemental Table 2) (FDR < 0.05). This signature was distinct from the previous two vascular profiles as determined by gene overlap, with one gene overlapping with the Wallgard et al. signature (*SLCO2A1*) and one gene overlapping with the vascular content signature (*CYP1A1*). Ingenuity Analysis identified top networks as cell cycle, cellular growth and proliferation, and lipid metabolism. This ‘activated endothelium’ signature was highest in basal-like tumors (*t*-test; *p* < 0.03 compared to claudin-low tumors) and strikingly under-expressed in normal mouse mammary tissues and monocultures of human ECs (Fig. 1C). Interestingly, recent immunohistochemical and magnetic resonance imaging studies have found that heightened vascular proliferation occurs in basal-like breast cancers [21, 22].

Lastly, we aimed to contrast the information provided by these genetic programs with a distant metastasis associated VEGF/hypoxia signature from Hu et al. [23]; this signature was the most highly expressed in basal-like (*t*-test; *p* < 0.02 compared to claudin-low tumors) and claudin-low tumors (Fig. 1D) and only had one gene overlap with any of the other signatures (PLOD1; vascular content).

### The tumor-activated endothelium signature identifies proliferating vasculature

**Fig. 2:**
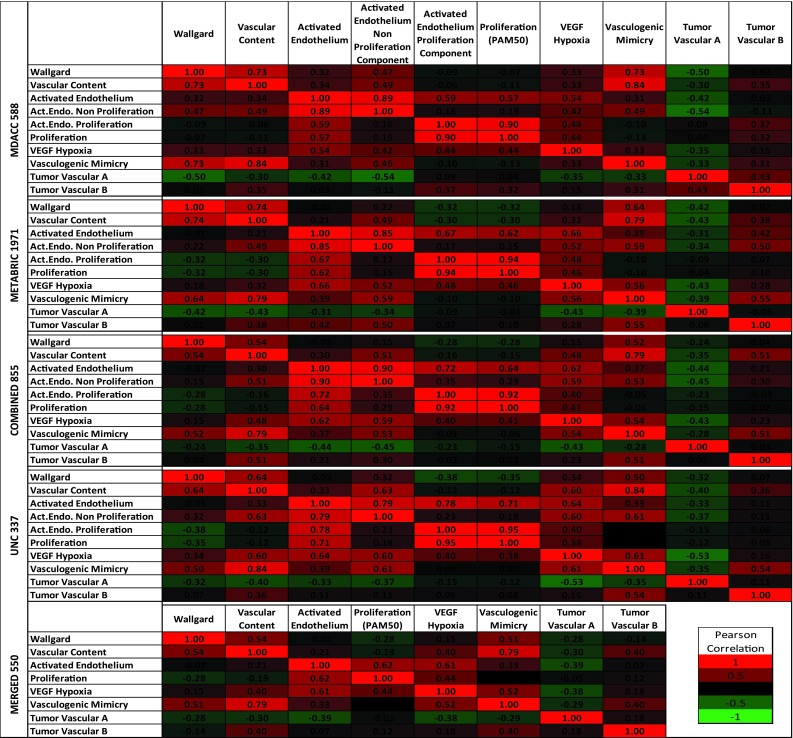
Assessment of the relatedness of vascular gene expression signatures. Shown are Pearson correlation coefficients of gene expression signatures from the five breast cancer datasets. Positive values are colored *red* and negative values are *green*

We next sought to directly compare each endothelial signature to several other vascular signatures that have been previously identified [17, 23–25]. Pearson correlations were determined between the various signatures and a known cell proliferation signature [12] using the same five breast cancer datasets. Other than the Tumor Vascular A signature [24], as a whole, most vascular signatures were positively correlated (Fig. 2). We also found that the activated endothelium signature showed a strong correlation with proliferation (0.57–0.71). We therefore ‘separated’ the proliferation component from the rest of the activated endothelium signature by identifying genes with a Pearson correlation greater than 0.5 to the proliferation signature [26]. This resulted in two distinct signatures: an ‘activated endothelium proliferation component’ and an ‘activated endothelium non-proliferation component’ (Supplemental Fig. 1). In each of the datasets tested, the vascular content, Wallgard et al., and vasculogenic mimicry signatures were all correlated (>0.5), suggesting that these three signatures were tracking similar biological processes. The VEGF/hypoxia signature showed positive correlations with activated endothelium; interestingly, both the proliferative and non-proliferative components of the activated endothelium signature had smaller Pearson correlations with the VEGF/hypoxia signature than the complete activated endothelium signature, indicating that the VEGF/hypoxia signature identifies both of these biological processes.

### Vascular signatures add metastasis predictive information within intrinsic subtypes

To determine if the expression of any of the vascular signatures correlated with increased metastasis in vivo, univariate Cox proportional hazards models analyses were performed for each signature on the five datasets (Supplemental Table 3). Several variables individually were able to significantly predict metastatic relapse in every dataset, including the luminal B and basal-like subtype status (as compared to luminal A), ER status, activated endothelium (including both the proliferation and non-proliferation components), VEGF/hypoxia, vasculogenic mimicry, and the 11-gene proliferation signature. We next used multivariate Cox proportional hazards models to determine if any of the vascular signatures provided additional prognostic information in addition to intrinsic subtype classification. In these analyses, each vascular signature was individually tested against the metastasis predicting information contained within intrinsic subtype status; note that only the vascular signature information is shown in Table 1. Interestingly, both the activated endothelium and VEGF/hypoxia signatures were the only two signatures that were found to be significant in all datasets. Therefore these signatures can be used to identify particularly aggressive subsets of tumors within a given intrinsic subtype (the full table including intrinsic subtype information is shown in Supplemental Table 4). These findings support the conclusion that the quality of endothelium and the specific heterotypic interactions between endothelium and epithelium are both important components of metastatic progression.

**Table 1:** Multivariate analyses of the vascular signatures tested individually against intrinsic subtype for metastasis prediction; tested in the five breast tumor datasets. Bolded signatures added metastasis predicting information to intrinsic subtype classification. Full table is presented in Supplemental Table 4

Signature	Combined 855		UNC 254		METABRIC 1971		MDACC493		MERGED 550	
	Hazard ratio	*p*-Value	Hazard ratio	*p*-Value	Hazard ratio	*p*-Value	Hazard ratio	*p*-Value	Hazard ratio	*p*-Value
Proliferation	1.428	5.26E-02	1.365	1.61E-01	**1.242**	**1.97E**-**07**	1.186	2.57E-01	**1.259**	**1.08E**-**02**
Wallgard	1.266	2.23E-01	0.913	8.51E-01	**1.222**	**2.02E**-**02**	**1.960**	**4.73E**-**02**	1.169	5.16E-01
Vascular content	**1.985**	**8.66E**-**05**	1.666	4.23E-01	1.192	2.07E-01	1.274	4.67E-01	**2.555**	**2.51E**-**04**
Vasculogenic mimicry	**1.450**	**8.80E**-**04**	1.300	4.53E-01	**1.278**	**1.70E**-**03**	**1.570**	**2.40E**-**02**	**1.720**	**8.50E**-**04**
***VEGF hypoxia***	***1.439***	***1.73E***-***06***	***3.255***	***2.16E***-***07***	***1.262***	***1.51E***-***07***	***1.609***	***1.54E***-***02***	***1.760***	***3.45E***-***06***
Tumor vascular A	1.263	2.65E-01	**0.128**	**1.46E**-**02**	**1.286**	**3.20E**-**02**	**0.291**	**6.12E**-**03**	0.879	6.42E-01
Tumor Vascular B	**1.967**	**1.08E**-**02**	1.081	8.96E-01	1.198	1.64E-01	0.803	6.07E-01	**2.220**	**1.65E**-**02**
***Activated endothelium***	***2.688***	***3.40E***-***03***	***18.406***	***4.67E***-***03***	***2.262***	***1.40E***-***03***	***7.460***	***2.29E***-***03***	***7.942***	***1.76E***-***05***
Act endo nonproliferation	**2.683**	**5.30E**-**03**	**11.021**	**2.62E**-**02**	1.473	1.38E-01	**11.232**	**4.82E**-**04**	–	–
Act endo proliferation	1.106	1.99E-01	1.614	5.96E-02	**1.305**	**4.51E**-**05**	1.035	8.01E-01	–	–

### Vascular signatures add metastasis predictive information to microvessel density scores

**Fig. 3:**
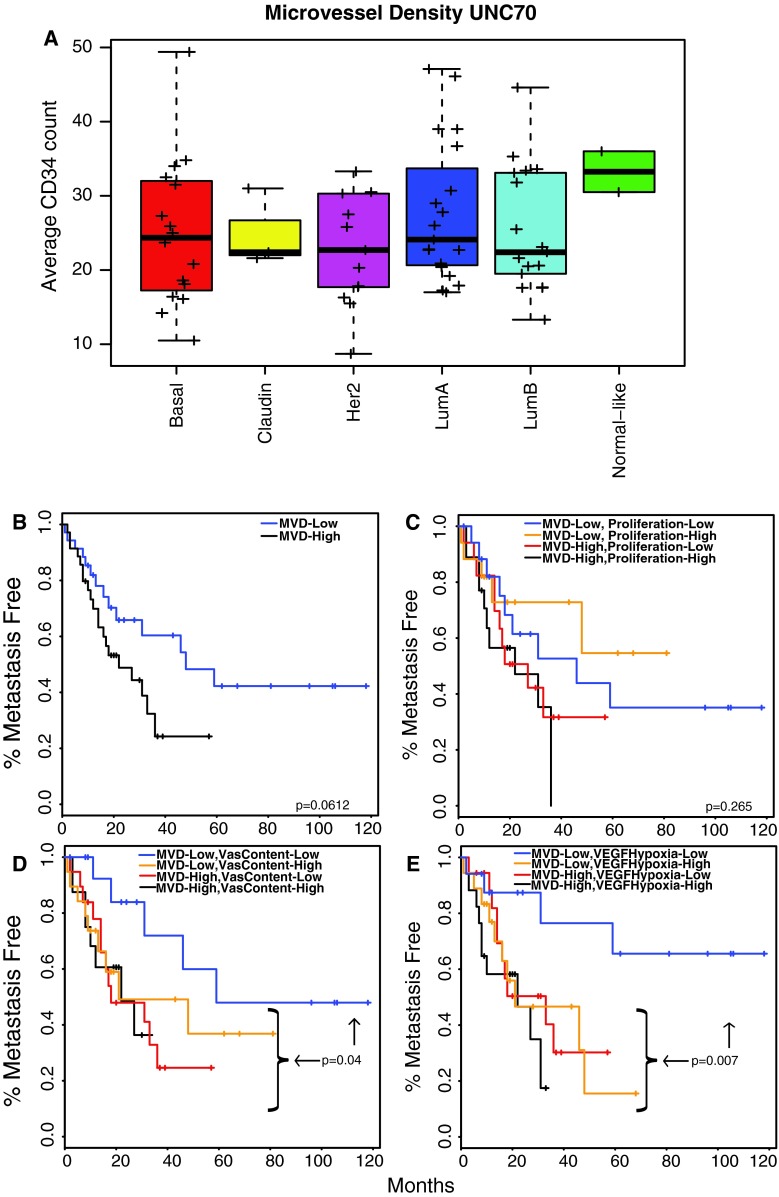
Gene expression signatures add prognostic information to immunohistochemistry defined microvessel density scores. **a** Box-and-whisker plots are shown for average microvessel density scores for 70 human breast tumors. **b–e** Kaplan–Meier plots for relapse free survival and log-rank test *p*-values. For testing more than one variable **c–e**, tumors were independently ranked from low to high signature score and then the two groups were combined, which yielded groups of not necessarily equal number that were reflective of the biology of the tumor. The *p*-value in **d** and **e** test the tumors with low signature scores for both variables against all other tumors

We next aimed to understand if any of the vascular gene expression signatures were a surrogate for the histology-based microvessel density (MVD) assay. Therefore, MVD scores were determined on a 70-tumor dataset that had also been subjected to gene expression microarrays (UNC70) (Supplemental Table 5). Although the sample set was small, on average MVD was similar across the breast cancer subtypes, except in the normal-like tumors which are comprised mostly of normal breast tissues (Fig. 3A). In this dataset, high MVD was significantly associated with decreased relapse free survival in a univariate analysis when tested as a continuous variable (*p* = 0.04), and was also trending towards significance in Kaplan–Meier analyses when the sample set was divided into halves based upon the rank order expression of this gene set (*p* = 0.06) (Fig. 3B). When tested in multivariate Cox proportional hazard models along with intrinsic subtype classification, MVD scores significantly contributed metastasis prediction information (*p* = 0.04).

Interestingly, additional multivariate analyses found that the gene signatures for vascular content, activated endothelium non-proliferation component, and VEGF/hypoxia all independently added metastasis-predictive information to the MVD scores (*p* < 0.05), unlike the proliferation related signatures (PAM50 11-gene proliferation signature and the activated endothelium proliferation component signature; *p* < 0.3). Similar results were also found with Kaplan–Meier plots shown in Fig. 3C–E. In these analyses, in contrast to proliferation which did not significantly help to stratify aggressive tumors (Fig. 3C), the additional prognostic information provided by the endothelial signatures when combined with MVD scores (Fig. 3D, E), inform us that using both the MVD scores and either of these two EC signatures are better than using either method individually.

### Claudin-low breast cancer cell lines have endothelial characteristics

**Fig. 4:**
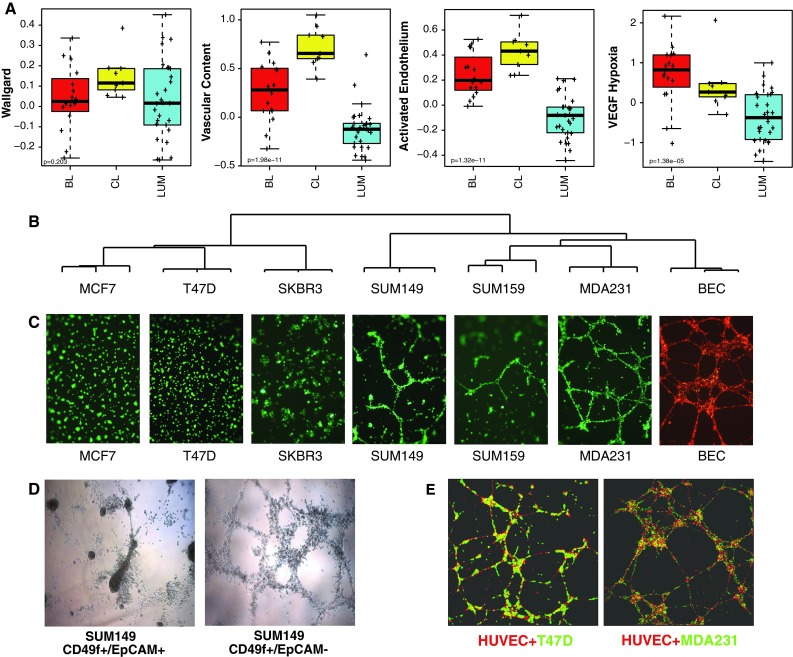
Gene expression and morphologic relatedness of endothelial cells and breast cancer cell lines. **a** Box-and-whisker plots of vascular signatures found in human breast cancer cell lines: BL; basal-like, CL; claudin-low, LUM; luminal. **b** Unsupervised hierarchical cluster dendogram of breast cancer cell lines and endothelial cells using all variably expressed genes (*n* = 12,644). **c** Picture of each cell line after 18 h of 3D culture (×5). **d** Pictures of the FAC sorted SUM149 cell line fractions after 18 h of 3D culture (×5). **e** Pictures of cocultures after 18 h of 3D culture (×5)

We next aimed to elucidate why claudin-low tumors had the highest expression of the gene signatures that were designed to measure total endothelial quantity: Wallgard et al., vascular content, and activated endothelium non-proliferation component (Fig. 1; Supplemental Fig. 1). We reasoned that high vascular signature scores could either be attributed to the amount of vasculature present in these tumors, or due to the extent in which claudin-low breast cancer cells express endothelial genes. Since, the MVD scores suggest similar amounts of vasculature across the subtypes as assessed histologically (Fig. 3A), we hypothesized that claudin-low tumor cells themselves may express these vascular cell associated genes. Therefore, we identified vascular signature scores for the human breast cancer cell lines presented in Neve et al. [2, 27.] and found that the vascular content gene expression signature was most predominately expressed in claudin-low as compared to basal-like (*p* < 0.01) or luminal (*p* < 0.001) breast cancer cell lines (Fig. 4A). To further test the hypothesis that claudin-low cell lines have endothelial cell characteristics, we performed unsupervised hierarchical clustering using gene expression data from six breast cancer cell lines representative of different intrinsic subtypes and blood vessel endothelial cells (BECs). Interestingly, clustering with all available expressed genes (12,644) (Fig. 4B), or the vascular content gene signature (not shown), showed that claudin-low cell lines (i.e. MDA-MB-231 and SUM159) are transcriptomically more similar to BECs than they are to other breast epithelial cancer lines.

Next, we aimed to understand if these expression similarities might also be manifested phenotypically, such as in tube/chord-like formation that occurs with ECs grown in three dimensional matrices [28]. To assess this phenotype, the six breast cancer cell lines and BECs were grown in Matrigel with EC media. The MDA-MB-231 cell line was striking in its resemblance to the BECs for tube/loop formation (Fig. 4C). The SUM149 cell line, which is frequently used as a model for inflammatory breast cancer, and SUM159 cell lines also exhibited tube formation, which was in contrast to the luminal (MCF7, T47D) and luminal/HER2-enriched (SKBR3) line that formed irregular spherical clusters.

Some breast cancer cell lines are cellularly heterogeneous and contain mixed populations of cells [2, 29], therefore, we sorted the basal-like SUM149 cell line into two distinct populations based on expression of EpCAM and CD49f [2, 30]. When grown on Matrigel, the EpCAM+/CD49f + fraction formed cluster-like structures while the EpCAM-/CD49f + fraction formed tube-like structures (Fig. 4D); interestingly, it is the EpCAM-/CD49f + fraction that shows the more claudin-low-like expression features [2]. To determine how different subtypes of breast cancer cells directly interact with ECs, we established a two-color three-dimensional co-culture model with cancer cells and HUVECs (Fig. 4E), or BECs (Supplemental Fig. 2). In co-culture, the luminal and HER2+lines tended to form cancer cell clumps on top of the EC loops, while the claudin-low lines interdigitated with the ECs.

Since claudin-low breast cancers and melanomas share extensive similarities in gene expression profiles [9, 31], we were curious if these tube-like structures were reminiscent of what has been termed ‘vasculogenic mimicry’ (VM), which was reported first in melanomas [25]. In vitro we saw no evidence of lumen formation, so it is unlikely such tube-like structures can actually support blood flow, but pseudo-vasculature has been interpreted as a survival strategy arising from genetically pliable tumors [32]. Interestingly, two genes reported to be involved in VM and pseudo-comedo formation, *Ang2/Angpt2* [33] and *Cox2/Ptgs2* [34], are also most highly expressed in claudin-low tumors (Supplemental Fig. 3). Glioblastoma stem-like cells have also been shown to exhibit similar phenotypic and functional features of ECs [35] and ovarian cancer has also been reported to exhibit VM [36]. Correspondingly, a VM gene expression signature [25], along with the other two new vascular signatures discussed above, were also the most highly expressed in the mesenchymal subtype of glioblastoma and ovarian cancer [37, 38] (Supplemental Fig. 4).

### High vascular permeability in claudin-low tumors

**Fig. 5:**
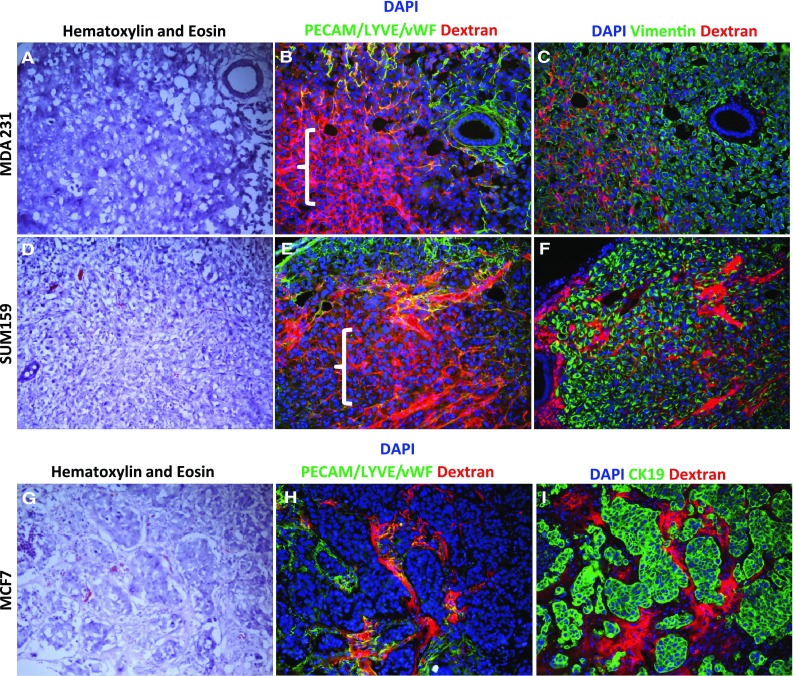
Identification of paracellular perfusion in claudin-low tumors. Texas Red Dextran (*red*) was injected into the circulation of mice bearing MDA231 (**a–c**), SUM159 (**d–f**), or MCF7 (**g–i**) cells grown as tumors in vivo. Serial sections for each tumor are shown. Hematoxylin and eosin staining **(a, d, g)**; pan-endothelial antibodies vWF/PECAM/LYVE (*green*), DAPI (*blue*), and dextran (*red*) **(b, e, h)**; vimentin or cytokeratin 19 cancer cell markers (*green*), DAPI (*blue*), and dextran (*red*) **(c, f, i)**. Brackets denote extensive dextran perfusion in the absence of vasculature. All images are ×20

Given the genomic and morphologic similarities of claudin-low cell lines and endothelial cell lines, we next aimed to determine if tubular structures were formed by claudin-low cancer cells (Fig. 5A–F), as compared to luminal cancer cells (Fig. 5G–I), growing in vivo. To identify if any tube-like structures were able to functionally perfuse blood, mice were injected intravenously with Texas Red labeled dextran 5 min before euthanasia [15]. When subjected to pan-endothelial antibodies platelet/endothelial cell adhesion molecule (PECAM), von Willebrand factor (vWF), and the lymphatic vessel endothelial hyaluronan receptor 1 (LYVE1) simultaneously, the claudin-low tumors were found to have  extensive perfusion of dextran through paracellular spaces (Fig. 5B, E). This heightened vascular permeability was not observed in the luminal MCF-7 model (Fig. 5H). Serial frozen sections that utilized the cancer cell markers vimentin (Fig. 5C, F) or CK19 (Fig. 5I) confirm that the dextran freely diffused throughout and around the claudin-low tumors but was largely restricted to the vasculature in luminal tumors.

## Discussion

The vasculature interacts directly with breast cancer cells and facilitates the growth and spread of nearly all human breast tumors. Since it is well documented that high microvessel density is associated with increased metastatic potential in many cancer types [6, 39, 40], which we also find herein for breast tumors, we hypothesized that variability in both the total amount of vasculature present, and the mechanisms used by different types of malignant breast cells to interact with ECs, may explain why the intrinsic subtypes metastasize at different rates and to different vital organs [9, 41, 42]. In these studies, we find that two independently derived gene signatures that measured the amount of tumor vasculature were consistently the most highly expressed in claudin-low tumors. Claudin-low breast cancer cell lines also expressed elevated levels of genes found in endothelial cells, exhibited endothelial-like morphologies when grown in three-dimensional matrices, and promoted vascular leakiness in vivo. These results provide evidence that the claudin-low cancer cells themselves have endothelial characteristics; qualities which may facilitate blood brain barrier penetration and metastasis to the brain [9, 43]. From these findings, we hypothesize that the subset of cells that most strongly express the claudin-low characteristics within a tumor are the cells that initiate tumor-endothelium interaction, the first step towards successful metastasis; the morphological differences observed with the different populations of SUM149 cells, which also have different growth rates, support this hypothesis. A recent paper supports this view and finds that MDA-MB-231 cells that adhere to the vasculature are Ki67-negative [44].

Previously, using different three-dimensional model systems, four distinct morphologies of breast cancer cell lines have been previously identified; Round, Mass, Grape-like, and Stellate [45]. Hierarchical clustering of gene expression microarrays from these cell lines found that their gene expression profiles largely correlated with their distinct morphologies and separate into luminal and basal-like branches of the dendogram. The Basal B subtype [27], which we refer to largely as claudin-low [2], is morphologically Stellate, whereas the luminal lines are classified as Round or Mass. The Stellate cell classification (BT-549, Hs578T, MDA-MB-231, MDA-MB-436) is particularly interesting for this manuscript since we have found that the MDA-MB-231 are claudin-low [2] in expression genotype, and form tubes/chords when grown in endothelial 3D conditions. The identification that claudin-low breast cancer cell lines present with vascular gene expression profiles and display endothelial-like morphology in 3D culture are significant because vascular-cancer mosaics have been found to increase vascular radioresistance [46]. Importantly, vascular characteristics of tumor cells have also been described in melanoma [25], ovarian cancer [36], Ewing sarcoma [47], and more recently in glioblastoma [35, 46]. Like the claudin-low breast cancer subtype that has endothelial/mesenchymal characteristics, both the ovarian and glioblastoma mesenchymal subtypes show highest expression of these vascular signatures, including a signature for vasculogenic mimicry. A previous report identified that 7.9 % of resected breast tumors exhibited vascular mimicry, which corresponded with an increased rate of hematogenous recurrence [33]. Interestingly, that report found that vascular mimicry containing specimens showed significantly higher *Angpt2* expression than non-vascular mimicry tumors. We evaluated *Angpt2* and found that this gene was highly expressed in claudin-low tumors. A different study found that cyclooxygenase-2 regulates vascular channel formation [34]. This gene is also highly expressed in claudin-low tumors and has been implicated in brain and lung metastasis [43, 48]. Both of these genes are known to be regulated by hypoxia. In cancer cells, intratumoral hypoxia generated by anti-vascular agents Sunitinib and Bevacizumab have been shown to increase the population of cancer stem cells [49], and it is the stem-like cells that are the ones most capable of exhibiting VM [35].

In conclusion, claudin-low tumor cells themselves exhibit vascular-like gene expression profiles in vivo and claudin-low breast cancer cell lines, and the claudin-low-like fractions within basal-like cell lines, also exhibit endothelial morphologies in vitro. These signatures of EC phenotypes predict the likelihood of breast tumor metastasis independent of tumor subtype, and also may have predictive potential for identifying patient cohorts that may respond to drugs targeting the tumor endothelium.

## Electronic supplementary material

Below is the link to the electronic supplementary material.

Supplementary material 1 (PDF 670 kb)

Supplementary material 2 (XLSX 47 kb)
