# Supplementary figures and images for: Endothelial-like properties of claudin-low breast cancer cells promote tumor vascular permeability and metastasis
Source: Clin Exp Metastasis. 2013 Aug 22;31(1):33–45. doi: 10.1007/s10585-013-9607-4 (PMC3892109; doi:10.1007/s10585-013-9607-4)

Supplemental Figure 1

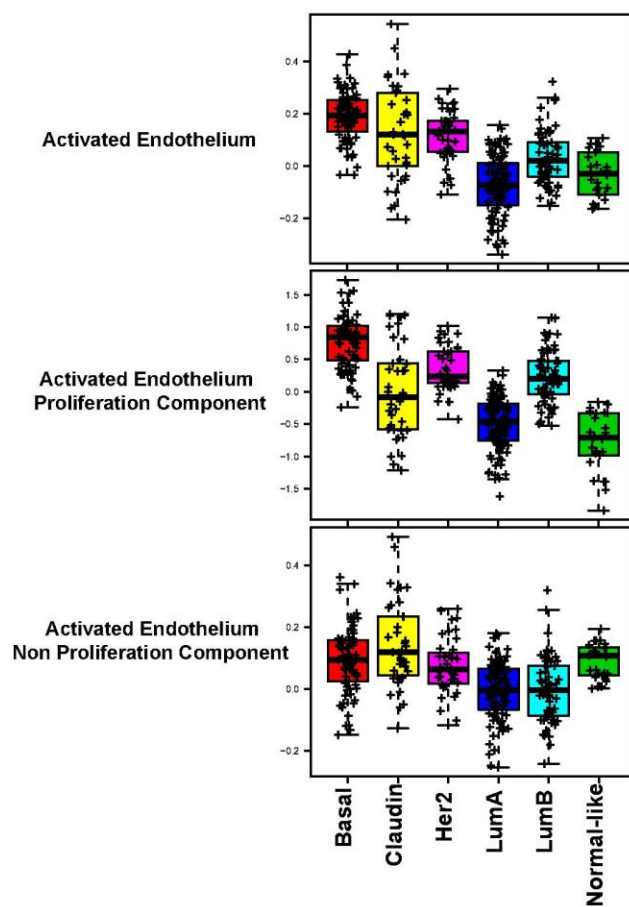

Supplemental Figure 2

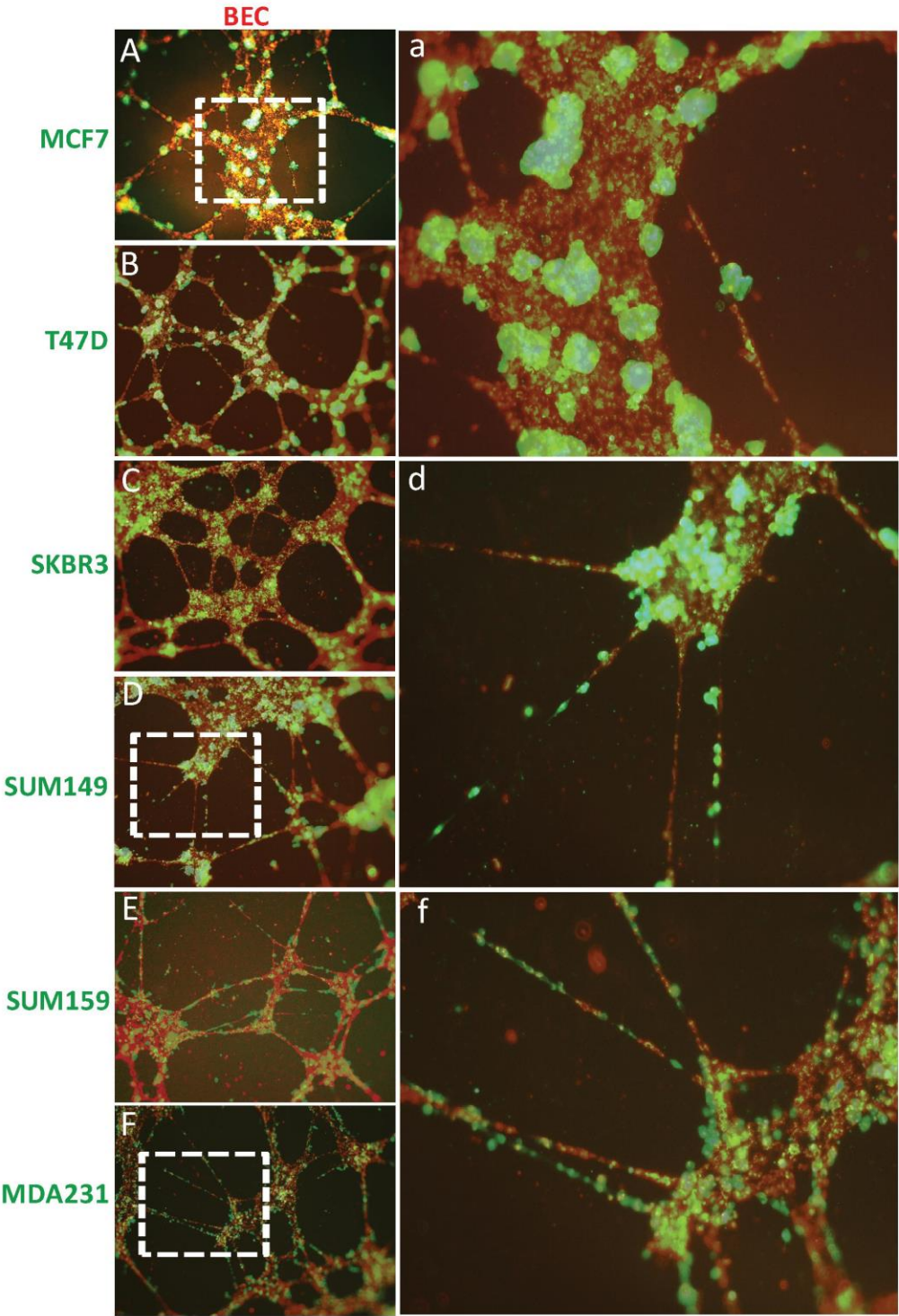

Supplemental Figure 3

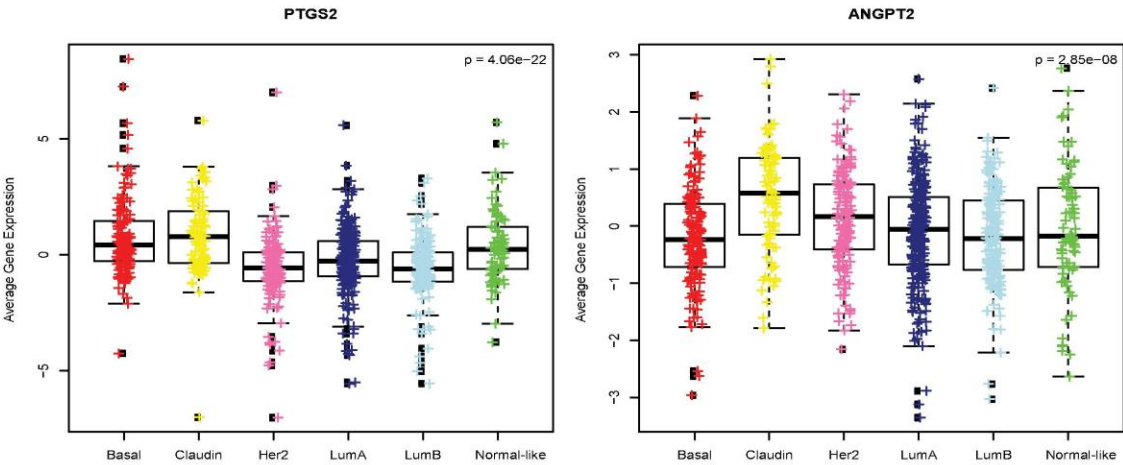

Supplemental Figure 4

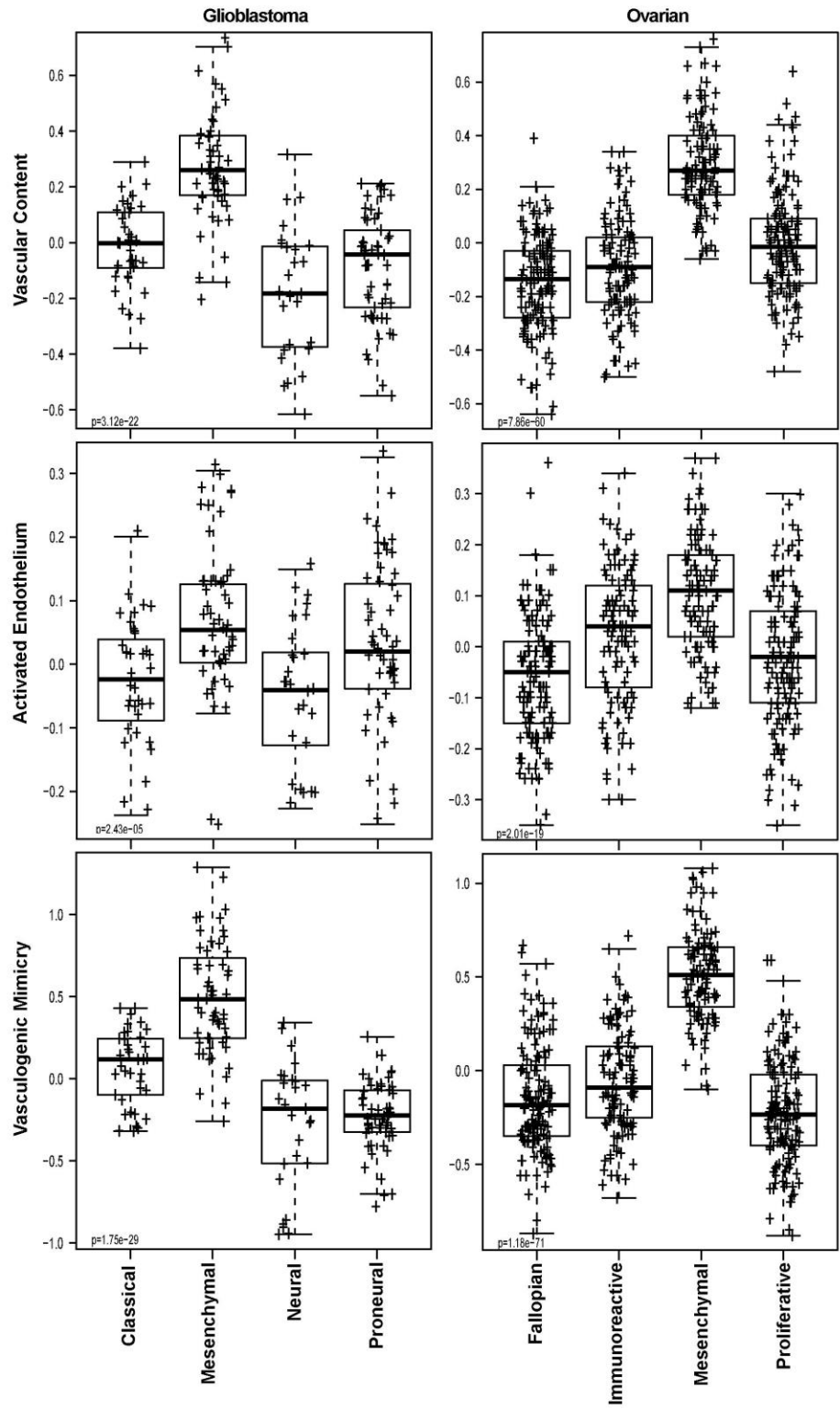

Supplement: Supplementary file 1 — Supplementary material 1 (PDF 670 kb) [file 10585_2013_9607_MOESM1_ESM.pdf]
